# Moving From CT to MRI Paradigm in Acute Ischemic Stroke: Feasibility, Effects on Stroke Diagnosis and Long-Term Outcomes

**DOI:** 10.1161/STROKEAHA.123.045154

**Published:** 2024-03-15

**Authors:** Costanza Maria Rapillo, Vincent Dunet, Silvia Pistocchi, Alexander Salerno, Vincent Darioli, Bruno Bartolini, Steven David Hajdu, Patrik Michel, Davide Strambo

**Affiliations:** Stroke Center, Service of Neurology, Department of Clinical Neuroscience (C.M.R., A.S., P.M., D.S.), University Hospital of Lausanne and University of Lausanne, Switzerland.; Neuroradiology Unit, Service of Diagnostic and Interventional Radiology, Department of Medical Radiology (V. Dunet, S.P., B.B., S.D.H.), University Hospital of Lausanne and University of Lausanne, Switzerland.; Emergency Department (V. Darioli) and Interventional Neuroradiological Unit, University Hospital of Lausanne and University of Lausanne, Switzerland.; Stroke Unit, Careggi University Hospital, Florence, Italy (C.M.R.).

**Keywords:** intracranial hemorrhage, ischemic stroke, neuroimaging, workflow

## Abstract

**BACKGROUND:**

The relative value of computed tomography (CT) and magnetic resonance imaging (MRI) in acute ischemic stroke (AIS) is debated. In May 2018, our center transitioned from using CT to MRI as first-line imaging for AIS. This retrospective study aims to assess the effects of this paradigm change on diagnosis and disability outcomes.

**METHODS:**

We compared all consecutive patients with confirmed diagnosis of AIS admitted to our center during the MRI-period (May 2018–August 2022) and an identical number of patients from the preceding CT-period (December 2012–April 2018). Univariable and multivariable analyses were performed to evaluate outcomes, including the number and delay of imaging exams, the rate of missed strokes, stroke mimics treated with thrombolysis, undetermined stroke mechanisms, length of hospitalization, and 3-month disability.

**RESULTS:**

The median age of the 2972 included patients was 76 years (interquartile range, 65–84), and 46% were female. In the MRI-period, 80% underwent MRI as first acute imaging. The proportion of patients requiring a second acute imaging modality for diagnostic ± revascularization reasons increased from 2.1% to 5% (*P*_unadj_ <0.05), but it decreased in the subacute phase from 79.0% to 60.1% (*P*_adj_ <0.05). In thrombolysis candidates, there was a 2-minute increase in door-to-imaging delay (*P*_adj_ <0.05). The rates of initially missed AIS diagnosis was similar (3.8% versus 4.4%, *P*_adj_=0.32) and thrombolysis in stroke mimics decreased by half (8.6% versus 4.3%; *P*_adj_ <0.05). Rates of unidentified stroke mechanism at hospital discharge were similar (22.8% versus 28.1%; *P*_adj_=0.99). The length of hospitalization decreased from 9 (interquartile range, 6–14) to 7 (interquartile range, 4–12) days (*P*_adj_=0.62). Disability at 3 months was similar (common adjusted odds ratio for favorable Rankin shift, 0.98 [95% CI, 0.71–1.36]; *P*_adj_=0.91), as well as mortality and symptomatic intracranial hemorrhage.

**CONCLUSIONS:**

A paradigm shift from CT to MRI as first-line imaging for AIS seems feasible in a comprehensive stroke center, with a minimally increased delay to imaging in thrombolysis candidates. MRI was associated with reduced thrombolysis rates of stroke mimics and subacute neuroimaging needs.

All patients with suspected stroke need brain imaging to differentiate ischemic from hemorrhagic stroke and from other neurological diseases that may present with focal neurological deficits.^1–6^ All these conditions require specific management whose benefits are highly time-dependent, especially in the case of recanalization treatments in acute ischemic stroke (AIS). Therefore, the emergency management of these patients requires to maximize the diagnostic accuracy and to minimize any possible delay. Given its short acquisition time and its widespread availability, computed tomography (CT)-based imaging has become the most widely used diagnostic study of suspected AIS. Despite being more time-consuming and less available, brain magnetic resonance imaging (MRI) offers several advantages over CT,^7^ including higher sensitivity in detecting early ischemic stroke, more precise localization of the ischemic lesion, more accurate detection of stroke mimics, likely better quantification of the ischemic core, and the capability to estimate the delay from stroke onset in patients with unknown onset.^8–10^ Currently, only a minority of stroke centers uses MRI as a first line tool for patients with AIS, and the data comparing MRI- with CT-first paradigm and its effect on patients’ outcome are limited.^8,9,11–13^

In our center, multimodal CT has been the first-line imaging modality for the assessment of all AIS from 2003 till May 2018, when we started using a new MRI machine integrated in the emergency department. This change provided us the opportunity to assess the transition from CT to MRI.

The aim of this quality assurance project of the treatment practice in our institution was to evaluate the MRI-based paradigm in comparison with the CT-based approach in term of (1) feasibility, effect on the diagnostic workflow, (2) diagnostic accuracy for AIS in the emergency setting and (3) effect on the subacute work-up in the stroke-unit, and (4) clinical outcome.

## METHODS

### Data Availability

The raw, anonymized data that support the findings of this study are available from the corresponding author upon reasonable request and after signing a data transfer and use agreement. If such data are used for a publication, its methods should be communicated, and internationally recognized authorship rules should be applied.

### Study Population

The study was conducted on the ASTRAL (Acute Stroke Registry and Analysis of Lausanne) that collects since 2003 all AIS admitted to the stroke unit and intensive care unit of the Lausanne University Hospital, presenting within 24 hours of stroke onset or last proof of good health as published previously.^14^ We excluded from the current analysis secondary transfers from other hospitals, patients with a brain imaging already performed before arrival at our hospital, and in-hospital strokes.

All consecutive patients who met the inclusion criteria, admitted between May 1, 2018 (date when the MRI machine in the emergency room [ER] became operational) and August 31, 2022 were included in the MRI-paradigm group. Patients during this period were kept in the MRI-paradigm group even if the initial imaging performed was CT, and reasons for not performing MRI as initial imaging were recorded. The CT-paradigm comparison group consisted of an equal number of consecutive patients admitted before April 30, 2018, resulting in a study period starting in December 2012. The details of variables collected for each patient are detailed in the Supplemental Material.

### Patients’ Clinical Assessment

All patients with suspected acute stroke admitted to the ER in Lausanne University Hospital are evaluated by an emergency physician and the on-call neurologist. Neurological evaluation, National Institutes of Health Stroke Scale assessment, and neuroimaging are performed as soon as possible and within 24 hours regardless of treatment eligibility in all suspected acute strokes (last proof of good health <24 hours). If the prehospital evaluation by the paramedic team indicates a thrombolysis or thrombectomy candidate, a stroke code is activated for immediate and simultaneous evaluation on arrival by on-call neurologist and emergency physician.

### Neuroimaging Protocol

Multimodal CT-based imaging was performed on a 64-multidetector CT scanner (LightSpeed VCT, GE Healthcare, Milwaukee, WI) between December 2012 and November 2015, or on a 256-multidetector CT scanner (Revolution CT, GE Healthcare, Milwaukee, WI) thereafter. CT imaging protocol, including noncontrast CT, CT-perfusion, CT-angiography, and postcontrast series, was performed in patients with suspected AIS as part of standard of care.^15^ The acquisition of this CT-based protocol including CT-perfusion takes 8 minutes.

For MRI imaging, we used a 3-Tesla scanner (Vida, Siemens, Erlangen, Germany). The following sequences are acquired (with corresponding acquisition time): sagittal T1 gradient echo (1′10″), axial diffusion (1′54″), axial 2-dimensional fluid attenuated inversion recovery (2′24″), axial T2 gradient echo (2′24″), time-of-flight angiography (6′12″), gadolinium-enhanced cervical magnetic resonance angiography (1′53″), postcontrast axial T1 gradient echo (1′05″), and perfusion weighted imaging (1′05″), as previously described.^16^ Apparent diffusion coefficient maps were also generated from diffusion weighted imaging sequence. The duration of this MRI protocol is 19 minutes. If the patient is eligible for intravenous thrombolysis (IVT), r-tPA (recombinant tissue-type plasminogen activator) is got ready in the MRI-room and administered immediately after diffusion weighted imaging, fluid attenuated inversion recovery, and gradient echo sequences are available to exclude intracranial hemorrhages.

### Outcomes Definition

To evaluate feasibility of the MRI-paradigm and its effect on the diagnostic workflow of AIS, we assessed the following outcomes.

- The proportion of patients undergoing any neuroimaging within 24 hours after stroke onset or last proof of good health
- In the MRI period only, the rates and the reasons for not attempting MRI as first imaging modality, and for obtaining an insufficient quality exam when it was attempted as first modality within 24 hours after stroke onset
- The delay in minutes from hospital arrival to start of brain imaging: door-to-imaging delay for the overall cohort, and for the potential IVT candidates: arriving within 3.5 hours after known onset and National Institutes of Health Stroke Scale ≥4
- Proportion of intracranial (± extracranial) arterial imaging at baseline with sufficient quality
- Proportion of perfusion imaging at baseline with sufficient quality
- The frequency and the reasons for performing a second acute imaging modality within 24 hours after stroke onset performed for diagnostic or acute treatment decisions (other than repeat imaging for worsening or routine postrevascularization control imaging)

As parameters of diagnostic accuracy for AIS in the emergency setting, we considered the following.

- The rate of stroke mimics treated by IVT, calculated as proportion over the total number of patients treated with IVT. For this specific analysis, the count of stroke mimics treated with IVT was added to the number of patients with AIS treated with IVT in each period. We considered stroke mimics cases with an initial diagnosis of ischemic stroke (prompting the administration of IVT), but a final diagnosis at discharge other than stroke, based on the revision of initial and repeated imaging (which usually included MRI), clinical findings suggestive of another cause, and negative work-up for major causes of ischemic stroke.
- The rate of missed strokes (stroke chameleons), calculated as proportion over the total number of AIS admitted in the same period. Stroke chameleons were defined as a failure to suspect a stroke on initial medical evaluation in the ER, or the incorrect exclusion of stroke diagnosis after the initial negative neuroradiological evaluation.^4^ The identification of stroke in chameleons was later made by board certified neurologists based on the clinical course and by repeat neuroimaging.
- The rate of missed intracranial bleeding on first imaging in IVT-treated patients

To assess the effect of the MRI-paradigm on the subsequent subacute work-up in the stroke-unit, we evaluated the following.

- The rate of patients with undetermined stroke mechanism at the end of acute hospitalization, with or without complete work-up, according to TOAST (Trial of ORG 10172 in Acute Stroke Treatment) criteria.^17^
- The proportion of patients needing any further neuroimaging beyond the initial (single or double) baseline imaging until the end of the acute hospital phase (subacute imaging).
- Length of hospitalization in the acute stroke unit.

As clinical outcomes, we assessed the following.

- The disability at 3 months, measured as the shift of the modified Rankin Scale (mRS) at 3 months. mRS at 3 months was assessed at the routine clinical examination in the outpatient clinic (or by a structured telephonic interview) by Rankin-certified personnel aware to acute imaging modality.
- Mortality at 7 days and at 3 months.
- Symptomatic intracranial hemorrhage (SICH) within 36 hours according to the ECASS-2 (European Cooperative Acute Stroke Study 2) definition.

### Statistical Analysis

Continuous and ordinal variables were expressed as medians (with interquartile range [IQR]), and categorical variables as absolute counts (with percentage), unless stated otherwise.

Initially we compared each outcome between MRI-paradigm and CT-paradigm groups, using unadjusted regression analyses to calculate crude odds ratio (OR) for binary outcomes and beta coefficients for continuous variables, along with 95% CI, and unadjusted *P* values. We did not consider meaningful to perform an adjustment for potential confounders for certain outcomes; therefore, we opted for univariable descriptive comparison, and calculated only crude ORs and unadjusted *P* values for the following outcomes: the proportion of patients undergoing any neuroimaging within 24 hours after stroke onset or last proof of good health, the proportion of arterial and perfusion imaging at baseline with sufficient quality, the frequency of second acute imaging modality within 24 hours after stroke onset, and the rate of missed intracranial bleeding on first imaging in IVT-treated patients.

For the other outcomes, including door-to-imaging delay, stroke mimic treated with IVT, stroke chameleon, undetermined stroke mechanism, length of hospitalization, repeated neuroimaging during stroke unit hospitalization, 3-month mRS, 7-day and 3-month mortality and SICH, we performed multivariable analyses. As a preliminary step, we initially assessed the presence and the shape of temporal trends throughout the study period for each of these outcomes. To do this, we used generalized additive models to fit smooth curves for each outcome with a cubic smoothing spline term for the time variable. We chose generalized additive model given their flexibility in fitting nonlinear patterns in the data, allowing us to identify trends over time that may not have been captured using traditional linear models. Depending on whether the outcome was continuous, binary, or multicategory, we respectively used additive quantile regression model, generalized additive models with binomial distribution, and generalized additive model with ordered categorical outcome. The time variable was obtained by dividing the study period into 10 consecutive periods with an equal number of consecutive patients in each period. The results of this time trend analyses were displayed graphically and by reporting the *P* value of the nonlinear term, and the effective df, which indicate the degree of nonlinearity of the curve (effective df=1 is equivalent to a linear relationship, effective df >1 and ≤2 is considered a weakly nonlinear relationship, and effective df >2 implies a highly nonlinear relationship).^18^ Subsequently, we added to the models for each outcome the imaging paradigm variable, together with the time trend variable (if significant at the *P*<0.05 level in the previous analysis), and potential confounders (selected based on clinical plausibility among demographics, clinical and radiological features), to obtain adjusted OR (or adjusted beta coefficients for continuous outcome variables), and 95% CI. For all analyses, *P*<0.05 were considered significant.

The Strengthening the Reporting of Observational Studies in Epidemiology method was applied to report results.

### Standard Protocol Approvals, Registrations, and Patient Consents

ASTRAL follows institutional regulations for clinical and research databases. All data collected stem from routine clinical and radiological management. Before analysis, the data were anonymized following the principles of the Swiss Human Research Ordinance. Given that only anonymized data were used, there was no need for ethical commission approval or patient consent according to the Swiss Human Research Act and the applicable data protection legislation. Also, the status of patient consent did not need to be considered because this was mainly a quality assurance project of the treatment practice in our institutions, falling outside the Human Research Act.

## RESULTS

The median age of the 2972 included patients was 76 years (IQR, 65–84), and 1361 (46%) were female. The full description of the study cohort is displayed in Table 1 and Table S1.

**Table 1.**
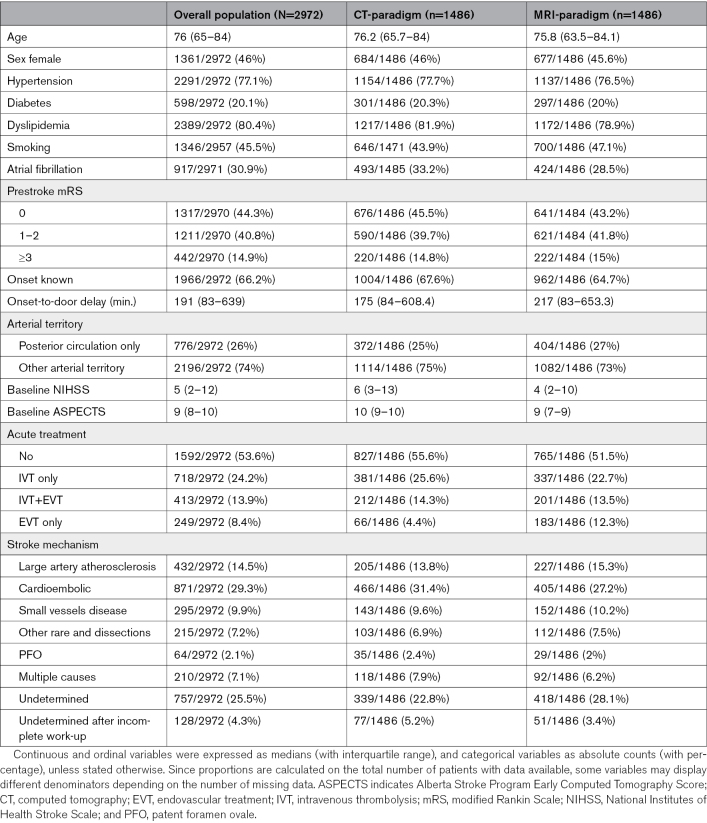
Patient Characteristics of the Overall Cohort and the 2 Groups of Interest

### AIS Diagnostic Workflow

**Figure 1.**
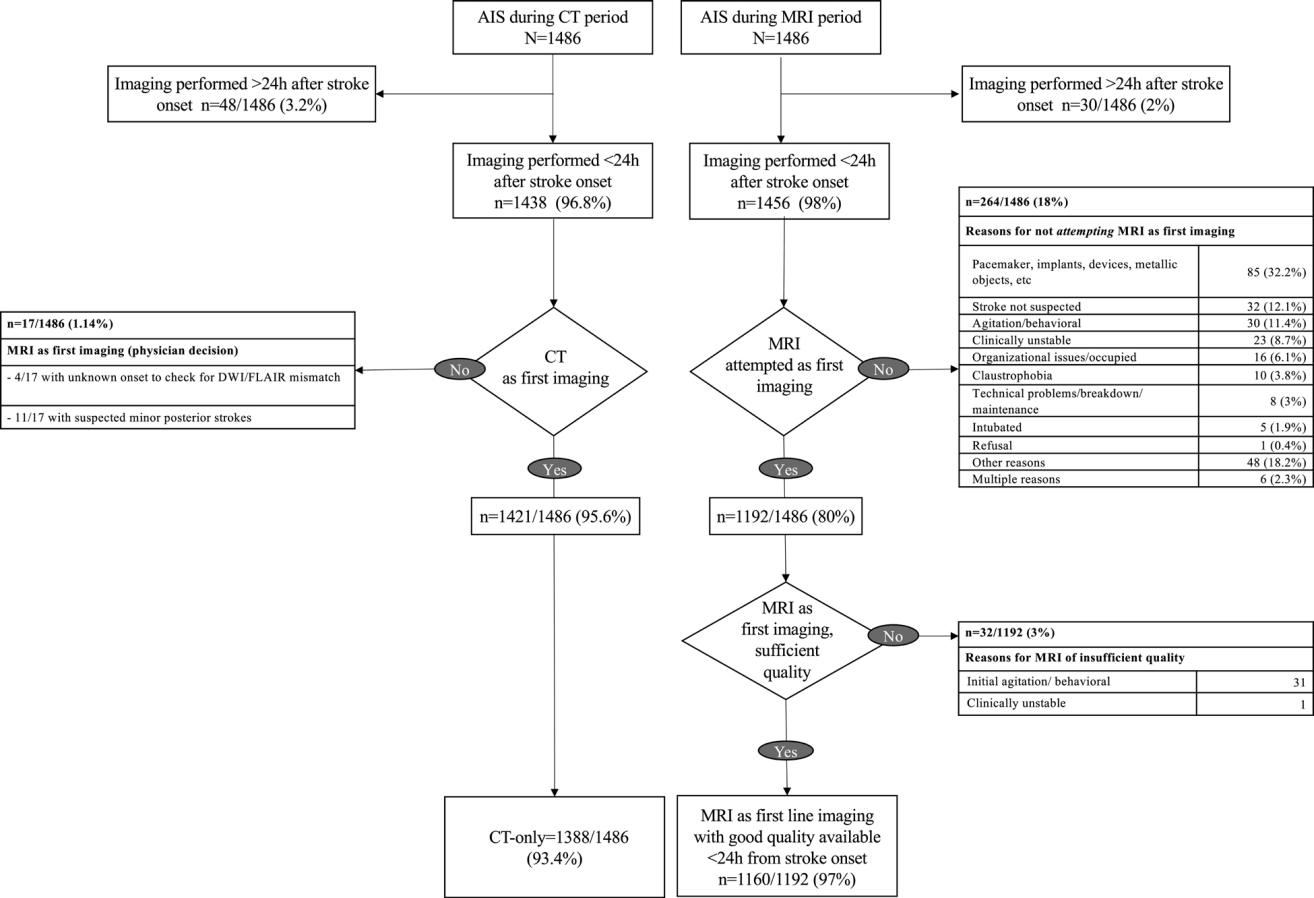
**Flowchart displaying the numbers and reasons for not obtaining the intended good quality study corresponding to the imaging paradigm period.** AIS indicates acute ischemic stroke; CT, computed tomography; and MRI, magnetic resonance imaging.

Among 1486 patients admitted during the MRI-paradigm (2018–2022), 98% (n=1456) had a brain imaging within 24 hours after stroke onset. This is slightly but significantly higher than the 96.8% rate of the CT-paradigm (OR_unadj_, 1.62 [95% CI, 1.02–2.57]; *P*_unadj_<0.05; Table 2). MRI was attempted as first imaging modality in 80% of patients (n=1192). The reasons for not attempting acute MRI are displayed in Figure 1. When MRI was attempted as the first imaging, 32/1192 patients (2.7%) had an exam which was incomplete or of insufficient quality (reasons detailed in Figure 1), and a CT had to be added in the acute phase. Among 1160 patients who underwent an initial acute MRI of sufficient quality during the MRI period, 65 (5.6%) were found to be negative for an acute ischemic lesion, despite the final diagnosis of ischemic stroke. Out of these 65 patients, 29 underwent a second MRI, and in 13 of them, an acute ischemic lesion was detected that had not been visible on the initial exam. In 25 out of the 65 patients (38%) with negative acute MRI, ischemic stroke was localized in the vertebrobasilar territory based on the clinical deficit or the results of follow-up MRI. The proportion of patients with a negative acute MRI was slightly higher in vertebrobasilar strokes compared with anterior circulation strokes (7.8% versus 4.9%, *P*=0.077).

**Table 2.**
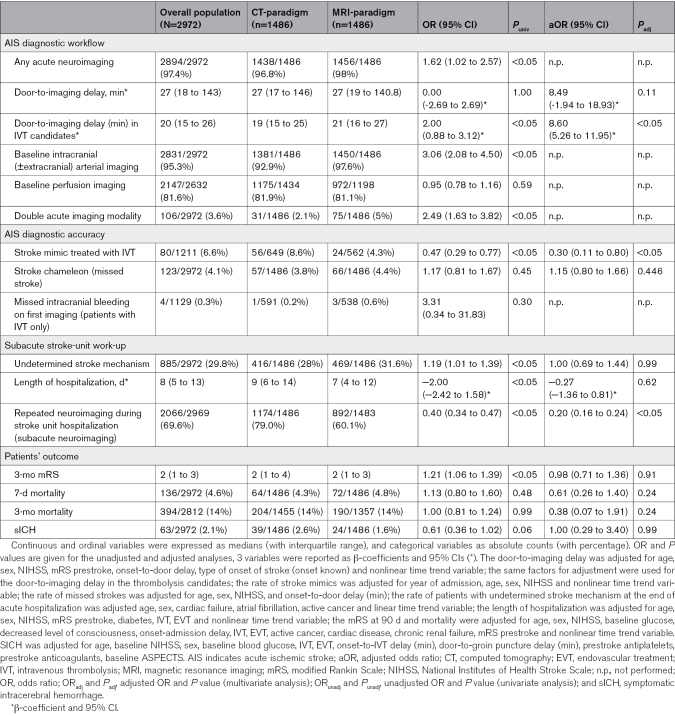
Outcomes of the Overall Cohort and the 2 Groups of Interest

**Figure 2.**
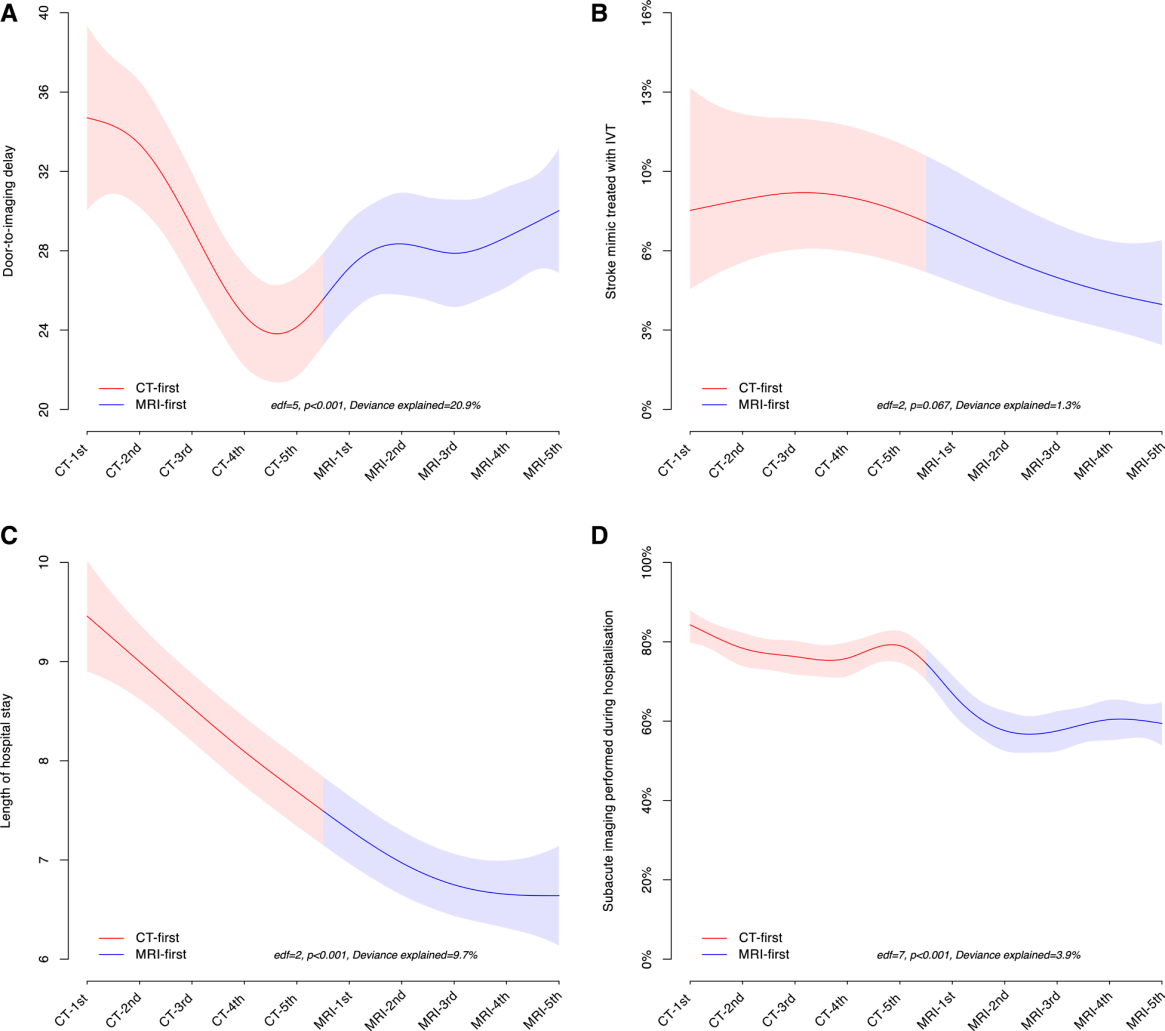
**Time trend analyses of 4 different outcomes, separated in groups of 300 patients. A**, Door-to-imaging delay; (**B**) rate of thrombolysis of stroke mimics; (**C**) length of hospital stay; and (**D**) rates of additional neuroimaging in the subacute phase of hospitalization. Red lines denotes computed tomography (CT) paradigm, blue lines: magnetic resonance imaging (MRI)-paradigm. CT 1st, 2nd, etc denotes first, second, etc groups of 300 patients in the CT paradigm. MRI 1st, 2nd, etc denoted first, second, etc groups of 300 patients in the MRI paradigm. edf indicates effective df; and IVT, intravenous thrombolysis.

We observed a significant nonlinear trend in the door-to-imaging delay over the study period (Figure 2A): during the CT-paradigm the delay progressively reduced, but since the beginning of the MRI-paradigm this trend inverted, indicating an increase in delay, although it never reached the values observed at the start of the study. Despite this nonlinear trend, the median door-to-imaging delay was 27 minutes in both periods, and in the multivariable analysis the imaging paradigm did not have a significant effect (β-coefficient, 8.49 [95% CI, −1.94 to 18.93]; *P*_adj_=0.11; Table 2). In the subgroup of potential IVT candidates (patients arriving to the ER within 3.5 hours from known onset and with baseline National Institutes of Health Stroke Scale ≥4), the median delay to imaging was 2 minutes longer in the MRI-paradigm as opposed to CT-paradigm: 21 minutes (IQR, 16–27) versus 19 minutes (IQR, 15–25; β-coefficient, 8.60 [95% CI, 5.26–11.95]; *P*_adj_<0.05).

The proportion of intracranial (±extracranial) arterial imaging with sufficient quality increased during the MRI-paradigm (MRI-paradigm, 97.6% versus CT-paradigm, 92.9%; OR_unadj_, 3.06 [95% CI, 2.08–4.50]; *P*_unadj_<0.05) while the proportion of perfusion imaging at baseline with sufficient quality was similar (Table 2).

During the CT-paradigm and the MRI-paradigm, respectively in 31 (2.1%) and 75 (5%) patients it was necessary to perform a second brain imaging modality for diagnostic or therapeutic reasons in the acute phase detailed in Table 3.

**Table 3.**
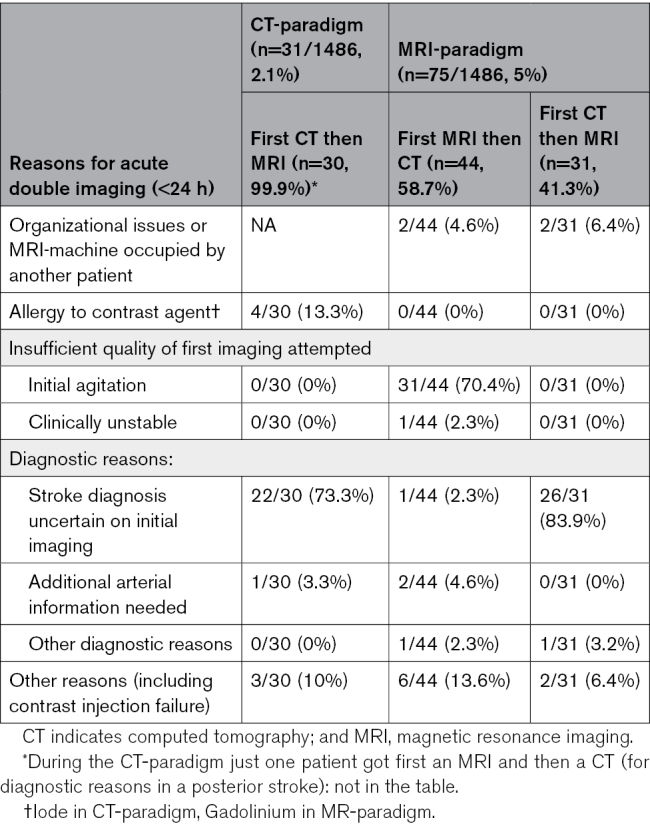
Proportion and Justification for Performing a Second Acute Imaging Modality for Technical or Clinical Reasons Within 24 Hours After Stroke Onset

### AIS Diagnostic Accuracy

Out of 1486 patients with AIS in each period, 593 and 538 were treated with IVT in the CT and in the MRI-paradigm, respectively. Additionally, 56 stroke mimics received IVT in the CT-paradigm and 24 in the MRI-paradigm. Therefore, the MRI-paradigm was associated with an ≈50% reduction of stroke mimics treated with IVT (MRI-paradigm, 4.3% versus CT-paradigm, 8.6%; OR_adj_, 0.30 [95% CI, 0.11–0.80]; *P*_adj_<0.05; Table 2). Figure 2B shows that, in addition to the decrease in the MRI-period, there was no significant further correlation between the inclusion period in the study and the proportion of IVT-treated stroke mimics.

The rates of AIS diagnosis initially missed (stroke chameleons) was similar in the 2 paradigms (MRI-paradigm, 4.4% versus CT-paradigm, 3.8%; OR_adj_, 1.15 [95% CI, 0.80–1.66]; *P*_adj_=0.45; Table 2; Figure S1A). The rate of missed intracranial bleeding on first imaging in IVT-treated patients remained unchanged (MRI-paradigm, 0.6% versus CT-paradigm, 0.2%; OR_unadj_, 3.31 [95% CI, 0.34–31.83]; *P*_unadj_=0.3; Table 2).

### Effect on the Subacute Stroke-Unit Work-Up and Length of Hospitalization

The proportion of patients with undetermined stroke mechanism at hospital discharge was not significantly different between the 2 periods (31.6 versus 28% in MRI versus CT-paradigm) after adjusting for age, sex, cardiac diseases, and cancer prevalence (OR_adj_, 1.00 [95% CI, 0.69–1.44]; *P*_adj_=0.99; Table 2; Figure S1B).

We found a significant trend in the length of hospitalization over the study period, characterized by a linear reduction that was consistent across both imaging paradigms and reached a plateau in the second part of the MRI period paradigm (Figure 2C). Although this resulted in a reduction in the median length of hospitalization from 9 days (IQR, 6–14) during the CT-paradigm to 7 days (IQR, 4–12) during the MRI-paradigm, the effect of the imaging paradigm did not reach statistical significance in the multivariable analysis (β coefficient, −0.27 [95% CI, −1.36 to 0.81]; *P*_adj_=0.62; Table 2).

The proportion of patients needing any further neuroimaging until the end of the acute hospital phase (subacute neuroimaging) decreased in the MRI-paradigm (MRI-paradigm, 60.1% versus CT-paradigm, 79.0%; OR_adj_, 0.20 [95% CI, 0.16–0.24]; *P*_adj_<0.05; Table 2; Figure 2D).

### Patients’ Outcome

The 3-month mRS was missing in 5.4% (160/2972) of patients. The difference in the 3-month functional outcome was not statistically significant between the 2 paradigms in the multivariable ordinal regression analysis (OR_adj_ for favorable mRS-shift of CT-paradigm versus MRI-paradigm, 0.98 [95% CI, 0.71–1.36]; *P*_adj_=0.91; Table 2; Figure S1C). We did not observe any statistically significant difference in mortality at 7 days and at 3 months nor in SICH (Table 2; Figure S1D through S1F).

## DISCUSSION

Our study showed the feasibility of using MRI as first imaging modality in all suspected AIS, since during the MRI-first paradigm 80% of patients effectively underwent MRI and 98% had an acute brain imaging exam. Compared with the CT-first paradigm, the MRI-first paradigm was associated with lower rates of stroke mimics treated with IVT and fewer repeated neuroimaging exams during the subsequent stroke unit hospitalization. However, MRI was associated with more double imaging modalities in the acute phase of stroke care. Although we also observed a slight increase of the door-to-imaging delay in potential IVT candidates, this was not significant in the overall population. The initial acute imaging modality did not affect the rate of missed strokes and missed intracranial bleeding, the rate of undetermined stroke mechanism at the end of the hospitalization, the length of hospitalization, or the 3 months functional outcome, mortality, and SICH.

A previous study on the feasibility of the MRI-first paradigm reported a lower rate of acute brain imaging (91%), but a higher rate of MRI as the first imaging modality (89.5%).^19^ In our cohort, the main reason for not attempting MRI was the presence of implanted devices, followed by patient’s agitation and clinical instability. The rate of double acute imaging during the MRI paradigm (5%) was higher than during the CT-first paradigm (2.1%). This occurred either from poor-quality initial MRI, mainly due to motion artifacts in agitated patients, requiring a CT after the initial interrupted MRI, or because MRI was added after an initial CT, mostly because of uncertainty of stroke diagnosis after a negative CT. Nonetheless, the MRI paradigm resulted in a reduction from 79% to 60% of subacute neuroimaging during the hospitalization phase, which largely compensated the increased double imaging in the acute phase and resulted in a lower overall number of brain imaging exams.

Although the median door-to-imaging delay in the overall AIS population was similar between MRI and CT paradigms, the introduction of the MRI did result in a reversal of the previously observed trend of progressive reduction in the delay. The longer delay to imaging initiation with MRI may in part result from the need to confirm of the absence of contraindications to MRI, such as the presence of pacemakers, metallic implants, or claustrophobia. Another factor is the patient preparation, which involves the removal of all metallic objects and the use of an MRI-compatible monitoring system. However, the median door-to-imaging delay of 27 minutes in the whole AIS population is comparable to other studies,^13,19^ and aligns with international guidelines recommending rapid imaging for patients with AIS.^20^ Even though these guidelines specifically address revascularization candidates, the same concept applies to all patients with AIS, and our study findings suggest that this can be achieved through both MRI and CT paradigms. In the subgroup of potential IVT candidates, we found a slight but statistically significant prolongation of the door-to-imaging delay of 19 versus 21 minutes, similarly to a previous observation.^21^ Ways to shorten door-to-imaging delays in a MRI-first paradigm include standard operating procedures to immediately identify MRI-contraindications before or at hospital arrival, like a checklist of MRI contraindications as routinely use at our hospital, the use exclusively MRI-compatible ECG-monitoring electrodes in the emergency phase, and conduct regular training sessions for the acute stroke pathway team. The duration of imaging acquisition with MRI can also be reduced by applying a minimal imaging sequence protocol that may omit either perfusion, cerebral time-of-flight, or contrast-based cervical magnetic resonance angiography in early patients with clear IVT or endovascular treatment indications.^22–24^

The finding of a significant reduction of IVT-treated stroke mimics associated to the MRI-first paradigm is not surprising given the better sensitivity of MRI compared with CT in detecting acute ischemic lesions.^2,25^ However, none of the previous studies comparing CT and MRI in AIS showed this effect of MRI.^8–13^

In addition, we did not find significant differences in rates of missed intracranial bleeding on first imaging in patients with IVT in a univariate comparison, consistent with the known accuracy of MRI to diagnose intracranial bleeding.^7,9^ The frequency of initially missed stroke (chameleons) did not change between CT and MRI-paradigm. This finding is not entirely surprising, given that one of the main reasons for chameleons is clinical under-recognition of stroke, not the type of neuroimaging.^4^

The proportion of undetermined stroke mechanisms was not affected by the acute imaging modality performed. It is possible that the high number of MRI performed during the stroke unit hospitalization may have masked the benefit of acute MRI on this outcome.

Regarding the length of hospitalization, we could not find a significant effect of MRI in reducing hospital stay. The observed time trend towards the reduction of hospital stay over the years, had already begun before the introduction of MRI and reached a plateau during the last years included in our study. However, we cannot exclude that the paradigm change participated in maintaining the temporal trend of hospital stay reduction, driven by the significantly lower number of repeated imaging in the subacute phase.

The 3-month disability and mortality were similar in the 2 study paradigms, similarly to previous studies comparing patients’ outcomes related to MRI in AIS.^12,13,26^ Other studies described a certain benefit of MRI in terms of in-hospital complications and mortality,^25,27^ even if results may be biased by baseline imbalances as patients undergoing MRI were younger and had less comorbidities.^27^

Our study has limitations. Given that this was a retrospective single-center quality assurance project conducted on a predominantly elderly, White cohort, its findings mainly apply to patients with these features and need to be confirmed in other populations. Furthermore, the patients were seen in a tertiary and university center stroke clinic, so there may be referral bias. The study conducted a time-period of 8 years when treatments and awareness of stroke have increased: stroke-care may have improved independently from the type of imaging used in the acute setting. We tried to minimize these potential effects by adjusting the analyses for a time variable. We did not perform any cost-analysis which could be a strong argument in healthcare system decisions, considering the budget when planning the first-imaging strategy for patients with stroke. Nevertheless, both the reduction of subacute imaging and the reduced rate of IVT-treated stroke mimics may be arguments in favor of MRI, indirectly reducing costs of acute stroke care. We studied only patients with confirmed AIS entered in the ASTRAL registry and did not assess all patients admitted to the ER with a suspected stroke and finally receiving an alternative diagnosis. Therefore, we could not assess the influence of MRI on these patients, including intracranial hemorrhages and stroke mimics not treated with IV thrombolysis. Finally, considering the wealth of results and information in the current article, we have not included separate analyses for the populations of patients treated with IVT or endovascular treatment assessing treatment-specific outcomes, such as door-to-needle time, door-to-puncture time, and SICH, which will be the subject of a separate analysis.

Strengths of the study include the systematic analysis of feasibility of an MRI-first paradigm for all arriving AIS patients accounting for many detailed outcome indicators. All previous studies comparing CT versus MRI-based imaging were focused on time metric, revascularization rates and functional outcomes.^8,9,12,13,28^ In addition, rather than comparing effectively performed CT versus MRI, we compared 2 imaging paradigms reflecting real clinical practice with its challenges and difficulties.

In conclusion, we could demonstrate the feasibility of MRI approach as first imaging modality for AIS. We showed several advantages of MRI over CT in term of acute diagnostic accuracy, mainly the significantly lower number of IV thrombolysis in conditions other than stroke, early identification of the ischemic lesion and reduced need to perform MRI during the subsequent hospitalization; the main disadvantage was the slightly longer delay to imaging in candidates to revascularization. Altough we showed some advantages of MRI over CT in term of acute diagnostic accuracy, mainly the significantly lower number of IV thrombolysis in conditions other than stroke thanks to MRI, the unchanged rate of patients with stroke initially not recognized in the ER (chameleons) underline the importance of an accurate clinical evaluation in the setting of acute stroke, which, at present, cannot be replaced even by the most sophisticated imaging available.

## ARTICLE INFORMATION

### Acknowledgments

The authors thank Melanie Price Hirt for English editing.

### Sources of Funding

Dr Rapillo acknowledges the European Academy of Neurology (EAN) for the 6-month research fellowship received.

### Disclosures

None.

### Supplemental Material

Supplemental Methods

Table S1

Figure S1

## Supplementary Material


