# Supplementary material for: Moving From CT to MRI Paradigm in Acute Ischemic Stroke: Feasibility, Effects on Stroke Diagnosis and Long-Term Outcomes
Source: Stroke. 2024 Mar 15;55(5):1329–38. doi: 10.1161/STROKEAHA.123.045154 (PMC11045552; doi:10.1161/STROKEAHA.123.045154)

SUPPLEMENTAL MATERIAL

Supplemental methods

Data collection in the ASTRAL registry

A large range of parameters were collected by stroke physicians and study nurses in a prespecified manner in ASTRAL during the acute hospital stay and then completed after discharge. The type and definition of variables collected in ASTRAL include demographics, medical history and cardiovascular risk factors, comorbidities, current medications, clinical symptoms and examination, process-oriented data, stroke localization, NIHSS on admission, physiological, metabolic, and hematologic parameters on admission, and acute and subacute brain and cervico-cerebral arterial imaging. We also recorded acute revascularization treatments, i.e. intravenous thrombolysis (IVT) and endovascular treatment (EVT), and clinical outcome at 3 months. Stroke mechanism was classified according to TOAST (Trial of Org 10172 in Acute Stroke Treatment) criteria [1]. For this study, we considered stroke without a determined cause at the end of acute hospitalization (with or without complete work-up) as "undetermined mechanism".

Table S1: additional baseline characteristics.
*The reader should consider in columns the different denominators depending on which missing data for each variable.

	**Overall population included (N=2972)**	**CT-paradigm (n=1486)**	**MRI-paradigm (n=1486)**
Coronary disease	562/2967* (18.9%)	275/1481 (18.6%)	287/1486 (19.3%)
Cardiac failure	141/2951 (4.8%)	77/1467 (5.2%)	64/1484 (4.3%)
Active cancer	171/2966 (5.8%)	78/1484 (5.3%)	93/1482 (6.3%)
Pre-stroke treatment			
**Antiplatelets**	1589/2932 (54.2%)	758/1447 (52.4%)	831/1485 (56%)
**Anticoagulants**	431/2969 (14.5%)	204/1485 (13.7%)	227/1484 (15.3%)
Leukoaraiosis	987/2158 (45.7%)	519/1326 (39.1%)	468/832 (56.2%)
Baseline decreased vigilance	294/2971 (9.9%)	175/1486 (11.8%)	119/1485 (8%)
Baseline systolic BP (mmHg)	151 (134-168)	152 (135.4-168)	150 (133-167)
Baseline diastolic BP (mmHg)	82 (72-92)	82 (71-92)	82 (73-92)
Baseline blood glucose	6.6 (5.7-8)	6.5 (5.7-8)	6.6 (5.8-8)

Figure S1. Time trend analyses of three different outcomes, separated in groups of 300 patients: A) missed stroke diagnosis (chameleons); B) undetermined stroke mechanism at the end of hospitalization; C) modified Rankin score at 3 months; D) mortality at 7 days; E) mortality at 3 months; F) SICH. Red lines = CT paradigm, blue lines: MRI-paradigm. CT 1st, 2nd etc. = first, second, etc. groups of 300 patients in the CT paradigm. MRI 1st, 2nd etc. = first, second, etc. groups of 300 patients in the MRI paradigm. edf= effective degrees of freedom.

Supplemental references

1. Adams HP, Jr., Bendixen BH, Kappelle LJ, Biller J, Love BB, Gordon DL, Marsh EE, 3rd. Classification of subtype of acute ischemic stroke. Definitions for use in a multicenter clinical trial. TOAST. Trial of Org 10172 in Acute Stroke Treatment. *Stroke*. 1993;24:35-41. doi: 10.1161/01.str.24.1.35

Supplemental Figure 1

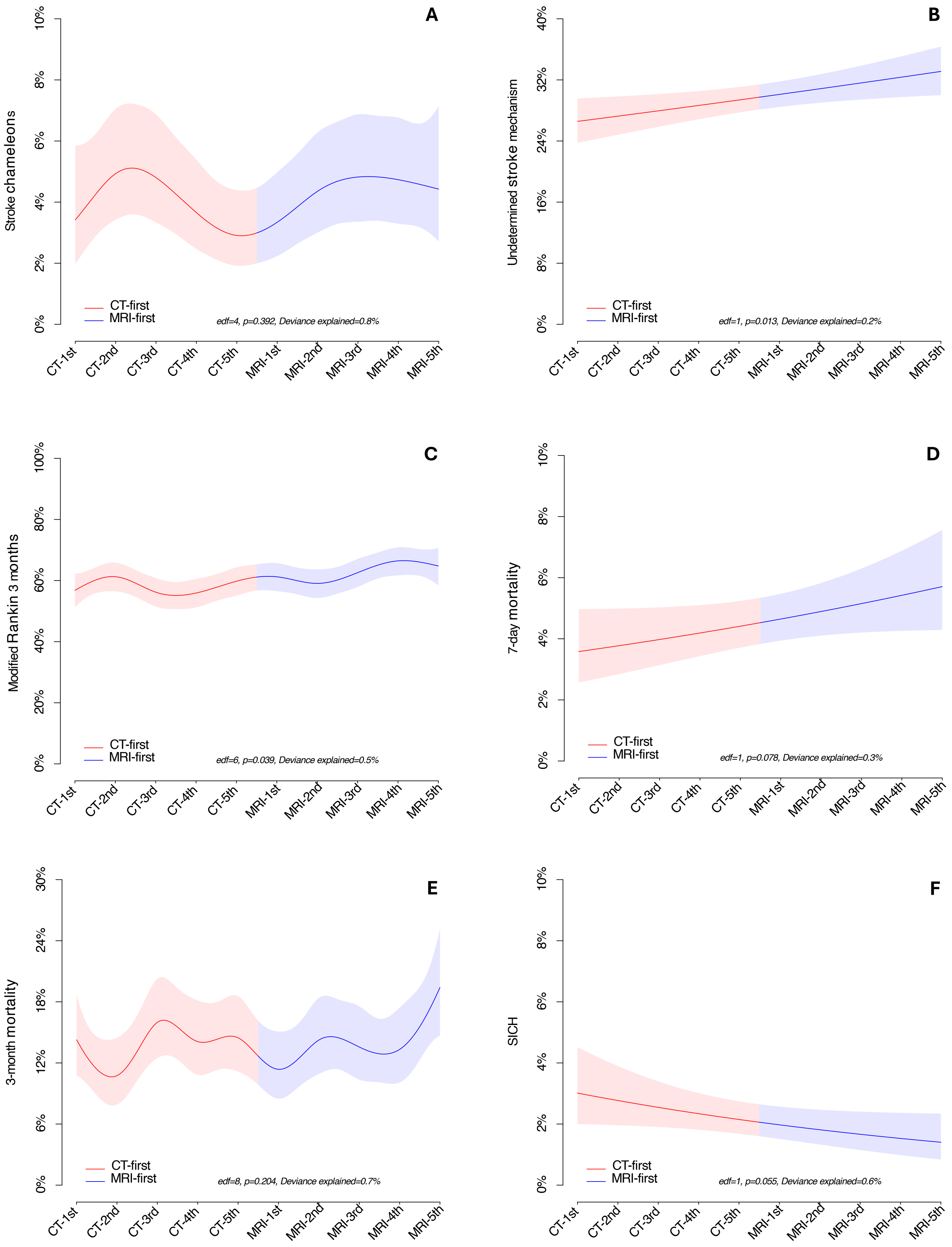

Supplement: Supplementary file 1 [file str-55-1329-s001.pdf]
